# A Blessing and a Curse? Political Institutions in the Growth and Decay of Generalized Trust: A Cross-National Panel Analysis, 1980–2009

**DOI:** 10.1371/journal.pone.0035120

**Published:** 2012-04-25

**Authors:** Blaine G. Robbins

**Affiliations:** Department of Sociology, University of Washington, Seattle, Washington, United States of America; Universita’ del Piemonte Orientale, Italy

## Abstract

Despite decades of research on social capital, studies that explore the relationship between political institutions and generalized trust–a key element of social capital–across time are sparse. To address this issue, we use various cross-national public-opinion data sets including the World Values Survey and employ pooled time-series OLS regression and fixed- and random-effects estimation techniques on an unbalanced panel of 74 countries and 248 observations spread over a 29-year time period. With these data and methods, we investigate the impact of five political-institutional factors–legal property rights, market regulations, labor market regulations, universality of socioeconomic provisions, and power-sharing capacity–on generalized trust. We find that generalized trust increases monotonically with the quality of property rights institutions, that labor market regulations increase generalized trust, and that power-sharing capacity of the state decreases generalized trust. While generalized trust increases as the government regulation of credit, business, and economic markets decreases and as the universality of socioeconomic provisions increases, both effects appear to be more sensitive to the countries included and the modeling techniques employed than the other political-institutional factors. In short, we find that political institutions simultaneously promote and undermine generalized trust.

## Introduction

Interest in trust has a long tradition in the social sciences and is considered a core dimension of social capital [1], [2], [3], [4]. For Georg Simmel, trust is “one of the most synthetic forces of society”[5]; for Robert Putnam, “honesty and trust lubricate the inevitable frictions of social life”[2]; and for the economist Kenneth Arrow, “virtually every commercial transaction has within itself an element of trust”[6]. Despite the prolonged interdisciplinary significance of trust, only in the last two decades have scholars of social capital considered the causes and consequences of an alternative yet closely related microfoundation known as generalized trust [7]. At the heart of this growing literature is the idea that generalized trust, or optimistic expectations about the trustworthiness of strangers, is important not just for the development of trust in known others but also for social order in general: it fosters cooperation across various political, economic, and social realms, and is thought to produce numerous normatively desirable outcomes such as economic growth [8], [9], life satisfaction [10], civic morality [2], and lower crime rates [11].

As one might suspect, the possible contextual sources of generalized trust are as equally diverse as its consequences [7], [12], [13]. For instance, numerous cross-national studies find political institutions to be closely related to generalized trust [14], [15], [16], [17], [18], [19], [20], [21]. Yet much controversy surrounds questions of which political institutions actually shape beliefs about the reliability of strangers in a given population. Some studies emphasize the effectiveness and quality of legal property rights institutions as the primary source of generalized trust [15], while others underscore the power-sharing capacities of the state [18], the government regulation of markets [22], or the universality of socioeconomic provisions [20]. But since cross-sectional studies populate this literature, it is plausible that issues of unobserved heterogeneity and simultaneity bias the results.

To address these problems, the present study fits fixed- and random-effects models to unbalanced cross-sectional time-series panel data from a wide variety of cross-national public opinion data sets administered in 74 countries between the years 1980 and 2009 to advance our understanding of how, why, and what political-institutional factors influence generalized trust through time. The panel structure of the data allows us to properly address issues of selection bias and unobserved heterogeneity and provides an opportunity to explore the underlying relationship between political institutions and generalized trust. This study is the first to engage in such an enterprise.

The findings show that generalized trust increases monotonically with the quality of property rights institutions, that labor market regulations increase generalized trust, and that power-sharing capacity of the state decreases generalized trust. While generalized trust increases as the government regulation of credit, business, and economic markets decreases and as the universality of socioeconomic provisions increases, both effects are more sensitive to influential cases, to modeling specifications, and to the types of countries included than the other political-institutional factors. Our results shed light on how political institutions simultaneously promote and undermine generalized trust.

### Political-institutional Foundations of Generalized Trust

Scholars who study the relationship between political institutions and generalized trust generally focus on four competing factors. First, most social scientists agree that our daily activities consist of situations in which we rely on actors we know very little about to help us accomplish tasks that are difficult to do alone, such as hire a lawyer to represent us in court, consult a dermatologist to screen us for skin cancer, or employ a computer scientist to design a webpage. It is generally recognized that a requisite amount of optimism concerning the trustworthiness of anonymous others must be in place in order to support such transactions; without this generalized trust, actors have little desire to risk exchange and good reason to avoid mutually beneficial lost opportunities. According to some scholars, the most effective means to foster generalized trust is to implement institutional incentives that induce actors we cannot readily judge or monitor to act in our interests and to behave in ways that we might call reliable [23], [24], [25]. In this way, institutional incentives create an environment where what is known by one actor about trustworthiness is also known by all other actors and that each actor holds optimistic expectations concerning the trustworthiness of anonymous others.

Among the variety of state enforcement mechanisms, rules of the game manifested as legal property rights are the most critical for the development of generalized trust [15], [20], [26], [27]. These institutional incentives go about promoting generalized trust indirectly and directly. Indirectly, institutional constraints buttress an environment where people can take risks and cooperate with others they know nothing about even if neither actor finds the other actor trustworthy or if neither actor holds optimistic expectations about the trustworthiness of strangers. If both actors exchange and benefit from this cooperative endeavor, then both actors update their beliefs concerning the other’s trustworthiness and update their beliefs about the trustworthiness of strangers [23], [24]. The more interactions an actor experiences like this, the more optimistic they become about anonymous others. Directly, institutional incentives allow individuals to feel safe and secure in their exchanges with others [28]. As long as these incentives provide the perception that institutional actors are able to minimize opportunism, then institutions foster the belief and expectation that anonymous others are reliable. If, on the other hand, institutional incentives are absent, then this fosters pessimistic expectations about a stranger’s trustworthiness. In either case, generalized trust is an indirect or direct result of institutional incentives.

Second, some social scientists suggest that political institutions used to promote reliability and trustworthiness ironically undermine the very things they are implemented to promote [29], [30], [31], [32]. Although this tradition generally focuses on all apparatuses of the state, we follow Aghion et al. [22] and restrict the undermining effects of government on generalized trust to the regulation of economic, business, and credit markets. Despite our restriction, at the heart of this tradition is the notion that political institutions undermine social cohesion and generalized trust via the mechanism of dependence: in the presence of centralized market regulations, individuals come to depend on those regulations and agents of the state, instead of each other, to promote mutually beneficial market outcomes. Cooperation and economic exchange, as a consequence, becomes less a result of interdependence and more a result of market regulation. This ultimately leads to the deterioration of community and civil society, fewer acts of altruism, a self-reinforcing dependence on state institutions to foster economic transactions, and a decline in generalized trust [22]. The implication is that generalized trust flourishes in the absence of government regulation, and that generalized trust wanes, although does not entirely disappear, in the presence of government regulation.

Yet not all market regulations undermine generalized trust. Efforts by the state to control labor markets with minimum wage laws, with prescriptions for collective bargaining, and with hiring and firing regulations, to name a few, increase generalized trust. In effect, these practices expand the rights of citizens and attempt to integrate the working class within the larger social system [33]. As Marshall [34] classically argued, these rights foster egalitarian economic systems and nationalistic social bonds that unify citizens. Both of which plant the seeds for generalized trust, especially among those who benefit from citizenship rights. In short, labor market regulations indirectly breed generalized trust by promoting egalitarianism and by fostering social integration.

Third, while some scholars emphasize political-institutional security and market regulation, others underscore the extent to which political institutions publicly allocate resources and are universally oriented [20], [35]. The argument here is that universal political institutions, such as welfare states, reduce the perception that government sides with certain economic actors over others. This helps generate the impression that each citizen has an equal opportunity for success and failure, creating a sense of shared fate, collective cohesion, and group solidarity that yields generalized trust [20], [36]. In other words, unfair governments foster economic inequality, inequality of opportunity, and unevenly distributed resources. When this occurs, social divisions and class hierarchies become ever more salient and perceptions of shared fate decline along with trust in generalized others [10]. The single way to overcome this outcome is with a government that equally divides public resources and enacts universal social welfare programs focused on leveling socioeconomic differences.

Fourth, some scholars suggest that the power-sharing capacity of the state is what fuels generalized trust [18], [37], [38]. Two possible mechanisms account for this effect. The first, cognitive inferences, suggests that certain kinds of prior experiences with other citizens is critical for the development of generalized trust [36]. In particular, if citizens are embedded within a partisan regime that is biased towards certain interests, then this regime fosters distrust among the disadvantaged and excluded groups. If, on the other hand, political institutions are non-partisan and welcome all interests to the political process, then generalized trust grows. Authoritarian and totalitarian regimes generally fall with the former, while democracies and systems of proportional representation generally fall with the latter. The second mechanism, socialization through transmission, refers to the capacity of political institutions to shape public opinion and build value-consensus [39]. The idea here is that habit-formative elements of power-sharing political institutions, such as a spirited associational life and consensus decision-making processes, permits the participation of all interests in the political process and, as a result, fosters generalized trust.

To summarize, the relationship between political institutions and generalized trust is complex and dynamic: some political-institutional elements foster generalized trust, while others undermine its development. These elements include property rights institutions, government regulation of economic, credit, and business markets, centralized regulation of labor markets, universal socioeconomic provisions, and power-sharing capacities of the state. Below we review the empirical evidence in support of these arguments.

### Political-institutions and Generalized Trust: The Evidence

Studies using cross-sectional data and ordinary least squares (OLS) regression tend to show that property rights institutions, court independence, contract enforcement, welfare state development, and democracy significantly and positively relate to aggregated micro-level public-opinion survey data of generalized trust [14], [17], [20], [21], [40], [41], while institutional transitions, corruption, and centralized market regulations tend to undermine generalized trust [20], [22]. Moreover, studies using ridge regression [42] or hierarchical linear models (HLM) in which observations are clustered into higher-level units typically parallel studies using OLS regression [15], [16], [19], [43], [44], [45], [46].

Although insightful, the association observed in these studies might represent a cause, an effect, or a common cause. A key assumption in both OLS and HLM is strict exogeneity: to the extent that generalized trust determines one or more of the independent variables in the equation (i.e., endogenizes the exogenous variables), then returned estimates will be biased and inconsistent, revealing an invalid directional association. While some studies attempt to overcome this bias with the method of instrumental variables using 2-stage least squares (2SLS) regression [47], [48], [49], [50], [51], the results are mixed as a consequence of empirically, historically, and/or theoretically suspect instruments [52], [53]. For instance, it is common to use gross domestic product [49], ethnolinguistic homogeneity [40], or legal origins [47] as instrumental variables for political institutions. While the instruments in these studies typically pass common instrument validity tests (e.g., the Cragg-Donald *F* statistic), rarely do these studies discuss and provide concrete evidence for independence (i.e., explain theoretically and empirically why an instrument is uncorrelated with unobserved causes of the outcome), the exclusion restriction (i.e., explain theoretically and empirically why an instrument does not have a direct effect on the outcome), or monotonicity (i.e., explain why the treatment is not available to those in the control group) [53]. In fact, we argue that the commonly used instrumental variables for political institutions thus far, such as gross domestic product, fail to satisfy any of these aforementioned requirements.

As a consequence, we still know very little about the political-institutional determinants of generalized trust. While research points to an association between these two factors, the causal effect might not run from institutions to generalized trust but from generalized trust to institutions [3], [18], [48], [50], [54] since the validity of instrumental variables for political institutions remains a contentious topic [55], [56], [57]. An alternative and some might say superior method is to employ modeling techniques that consider time [58]. Yet only one study in the literature has modeled such a relationship [18]. Although foundational, this study is not without shortcomings: it employed a small sample of 46 countries where maximum-likelihood missing value techniques were used on half of the sample, which can bias results [59]; it used cross-lagged structural equation panel models that did not account for time-invariant unobservables; and it explored only one element of political institutions, namely democracy.

In short, while these findings provide important insights into how and why political institutions simultaneously promote and undermine generalized trust, many questions remain. In particular, these studies show that time must be taken seriously as we assess the crowding-in and crowding-out effects of political institutions on generalized trust; that is, nearly all of the studies outlined above neglect to examine how changes in political institutions affect generalized trust. The goal of the present study is to address these remaining issues. We do so using fixed- and random-effects models that control for unobserved heterogeneity and address simultaneity.

## Methods

### Ethics Statement

All data used for the present study is secondary and publicly available. Human subjects were not directly contacted or surveyed by the author. The study was approved by the Human Subjects Division of the author’s university.

### Operationalization

We measure generalized trust by aggregating answers to the following binary question: “Generally speaking, would you say that most people can be trusted or that you need to be very careful in dealing with people?” In other words, it is the proportion of respondents–multiplied by 100–who say that most people can be trusted (ranging from 0 to 100). While this operationalization is not without criticism [60], [61], [62], [63], [64], we nevertheless rely on this measure for three reasons. First, scholars across various disciplines use it on a consistent basis [2], [10], [14], [18], [21], [47], [65], which facilitates cross-study comparisons. Second, recent research finds that in non-Confucian countries this operationalization of generalized trust sufficiently captures the notion that respondents think about people they do not know personally and have not yet met [66], [67]. Third, it is the only operationalization of generalized trust that spans the various time-series public opinion data sets we use. The unbalanced panel data on generalized trust are drawn from six waves of the World Values Survey (1981–1984, 1989–1993, 1994–1998, 1999–2004, 2005–2009), four waves of the Latinobarometer (Bolivia 1996, 2000, 2005; Brazil 2000; Columbia 2000; Costa Rica 1996, 2000, 2005; Dominican Republic 2004, 2005; Ecuador 1996, 2000, 2005; El Salvador 1996, 2000, 2005; Guatemala 1996, 2000; Honduras 1996, 2000, 2005; Nicaragua 1996, 2000, 2005; Panama 1996, 2000, 2005; Paraguay 1996, 2000, 2005; Uruguay 2000; Venezuela 2005), four waves of the Afrobarometer (Algeria 2006; Botswana 1999, 2005; Namibia 1999, 2005; Tanzania 2005; Zambia 2001), four waves of the Asiabarometer (Bangladesh 2005; Pakistan 2005; Philippines 2007; Singapore 2006; Sri Lanka 2003, 2005), the 1986 Eurobarometer 25 [8], and the 2008 European Values Study Wave IV. All generalized trust data are frequency weighted when available (e.g., WVS S017).

Data on political institutions are based on the following: (a) legal structure and security of property rights, (b) state regulation of credit, labor, and business, and (c) size of government measures drawn from the Economic Freedom of the World Dataset [68]. All three measures are used to capture legal property rights, government regulation of markets, and universality, respectively, and range from 0 (‘no economic freedom’) to 10 (‘total economic freedom’). Following prior research [15], [42], [69], we include a squared polynomial term for legal structure and security of property rights. To measure labor market regulations, we use the worker’s rights variable from Cingranelli and Richards [70]. To operationalize power-sharing capacity, we standardize and sum the following four measures: the political rights measure from United Nations Freedom House web resources, the power-sharing regime measure from Norris [71], the executive authority measure from the Polity IV Project, and the democracy measure from the Polity IV Project (α = .95) (note: greater values indicate greater power-sharing capacity).

We also control for a number of other factors that might confound the relationship between political institutions and generalized trust [10], [14], [72], [73]: first, we include income inequality based on the gini coefficient drawn from various sources (see Table S1); second, we include a measure of ethnolinguistic homogeneity taken from the *Encyclopedia Britannica* and CIA World Factbook, which consists of the percent largest ethnic and linguistic groups in a country summed and divided by two (α = .73); third, we include the natural log of gross domestic product per capita (constant year 2000 US$) from the World Bank to measure economic development and modernization; and fourth, we include a number of time-invariant variables that control for factors related to a country’s culture and values [49], [74], [75], [76], [77]–these include variables for countries with absolute or constitutional monarchies (Monarchy); for countries with Scandinavian cultural heritages such as Denmark, Finland, Iceland, Norway, and Sweden (Nordic); for a country’s average coldest month of the year in Celsius scale (Temperature); for license to pronoun-drop in the official language of a country (Pronoun-Drop); and for countries with former Marxist-Leninist governments (Former Communist) (for our sample, these countries include Albania, Bulgaria, Croatia, Czech Republic, Estonia, Hungary, Latvia, Lithuania, Poland, Romania, Russian Federation, Slovakia, Slovenia, and Ukraine).

All of these time-invariant controls consistently relate to, or have been used as instrumental variables for, generalized trust in prior research. We thus expect countries with constitutional or absolute monarchies and countries with Scandinavian cultural heritages to have higher levels of generalized trust than other countries. As Bjørnksov [49], [50], [72] has argued, monarchies engender national identity, collective unity, and social stability in a country that ought to foster generalized trust even in the face of economic and political cleavages, while countries with Scandinavian cultural heritages should promote generalized trust as a result of their economic, political, and social exceptionalism [14]. Following Bjørnksov [50], we also expect countries with extremely cold winters to exhibit high levels of generalized trust as harsh winters supposedly foster greater interdependence among strangers than countries with milder winters where individuals can rely on their immediate family and friends for survival. We also expect countries with official languages that permit dropping of the personal pronoun to exhibit lower levels of generalized trust than countries with official languages that forbid such practices: dropping the first person pronoun is typical of cultural traditions that place greater emphasis on collectivism, while forbidding first person pronoun drop is typical of cultural traditions that place a greater emphasis on individualism [48], [77]. Finally, generalized trust should be lower in former Marxist-Leninist governments as a result of (a) the economic and political dismantling within these countries after the fall of communism, and (b) the oppressive behavior of the former communist dictatorships [78], [79].

To reduce problems of simultaneity, all independent variables are lagged period *t*–*k* and correspond to the lagged *t*–*k* generalized trust survey year. See Table S1 for a description of the data sets and Table 1 for descriptive statistics.

**Table 1:** Description of variables and summary statistics.

Variables	Unit	Mean	SD	Min	Max
Generalized trust	Proportion of sample who believe that others can be trusted.	29.79	16.12	2.81	76.12
Legal property rights	10 = Property protection to 1 = no property protection.	6.47	1.69	2.70	9.60
State regulations	10 = No market regulation to 1 = complete regulation.	5.81	1.02	2.50	8.80
Worker’s rights	2 = Worker’s rights fully protected to 0 = severely restricted.	1.41	0.79	0.00	2.00
Size of government	10 = Market allocation of resources to 1 = Government allocation.	5.46	1.77	1.60	9.10
Power-sharing capacity	Standardized index (greater values equal greater power-sharing).	0.31	0.70	−2.20	0.77
Income inequality	Absolute inequality from 0–100.	37.11	11.16	20.70	74.33
Ethnolinguistic homogeneity	(% largest ethnic group + % largest linguistic group)/2.	81.57	16.28	29.30	100
ln(GDP)	ln(gross domestic product per capita, constant year 2000 US$).	8.62	1.26	5.65	10.51
Monarchy	1 = Monarchy, 0 = otherwise.	0.27	–	0	1
Nordic	1 = Denmark, Finland, Iceland, Norway, and Sweden, 0 = otherwise.	0.09	–	0	1
Temperature	Average temperature (Celsius) in the coldest month of the year.	7.08	9.92	−11	27
Pronoun-drop	1 = license to pronoun-drop in the official language, 0 = otherwise.	0.66	–	0	1
Former communist	1 = former Marxist-Leninist states, 0 = otherwise.	0.17	–	0	1

No. countries  = 74, No. observations  = 174 for all variables.

### Model Specification

In our first set of estimates, we model generalized trust as a function of the political-institutional variables and controls by pooling the time-series of the country sample and using OLS regression (model not shown formally). We then model generalized trust using random- and fixed-effects estimation techniques. The random-effects estimation is modeled as follows:



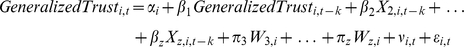
where *i* represents each country and *t* represents each time period (with t = 1–6 waves); *GeneralizedTrust_i,t_* is the generalized trust dependent variable for country *i* at period *t*; *GeneralizedTrust_i,t–k_* and *X_z,i,t–k_* are respectively generalized trust and time-variant predictors for country *i* during period *t–k* where *k* is the most adjacent period to *t*; *W_z,i_* are time-invariant predictors for country *i*; *β_z_* are the coefficients for the time-variant predictors; *π_z_* are the coefficients for the time-invariant predictors; *α_i_* represents the between-country constant term, *ν_i,t_* is the between-country error term, and *ε_i,t_* is the within-country error term.

Random-effects estimation techniques assume that the variation across entities is random and uncorrelated with predictors in the model. The advantage of random-effects estimation is the ability to include time-invariant regressors such as Scandinavian cultural heritage. But if *ν_i,t_* is correlated with the predictors in the model, then the random-effects estimates are biased and inconsistent. This would suggest the use of fixed-effects estimation, which is modeled as follows:
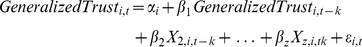
where *i* represents each country and *t* represents each time period (with t = 1–6 waves); *GeneralizedTrust_i,t_* is the generalized trust dependent variable for country *i* at period *t*; *GeneralizedTrust_i,t–k_* and *X_z,i,t–k_* are respectively generalized trust and time-variant predictors for country *i* during period *t–k* where *k* is the most adjacent period to *t*; *β_z_* are the coefficients for the time-variant predictors; *α_i_* represents the country-specific constant term and *ε_i,t_* is the error term.

We estimate our models using fixed-effects to allow for non-independence of observations within countries and to examine variation within countries and thus control for unobserved heterogeneity between countries. In other words, with fixed-effects estimation we control for time-invariant country-specific unobserved confounding variables. For instance, countries vary according to their legal origins, be it common, civil, or Islamic law that indirectly influence the development of generalized trust [47]. This explanation, however, emphasizes variance between countries, not within. Fixed-effects estimation controls for such country-specific explanations. Thus, unobserved variables do not change over time with fixed-effects estimation and, as a result, any changes in generalized trust must be due to predictors in the model and not due to time-invariant characteristics such as culture.

## Results

**Figure 1:**
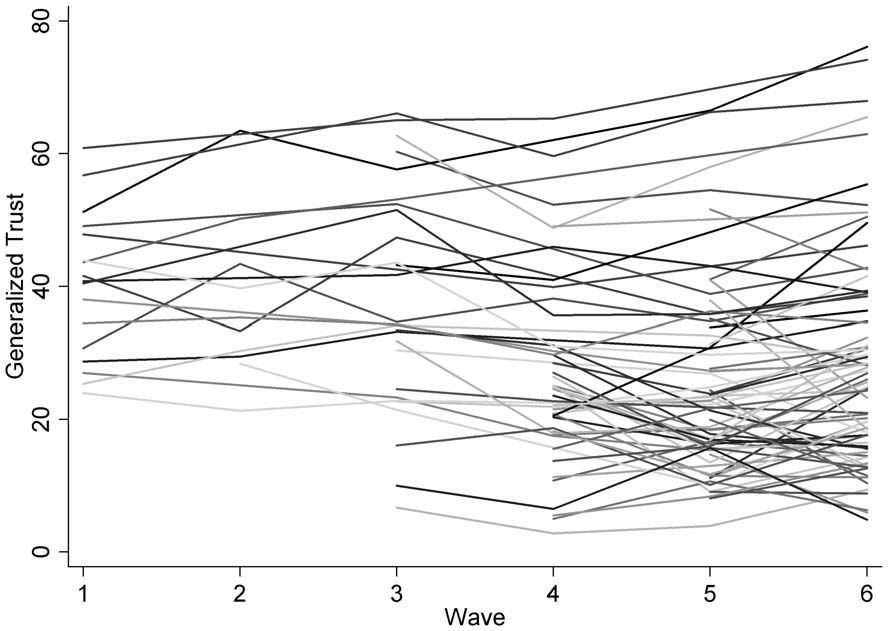
Generalized trust in 74 countries, 1980 to 2009.

**Figure 2:**
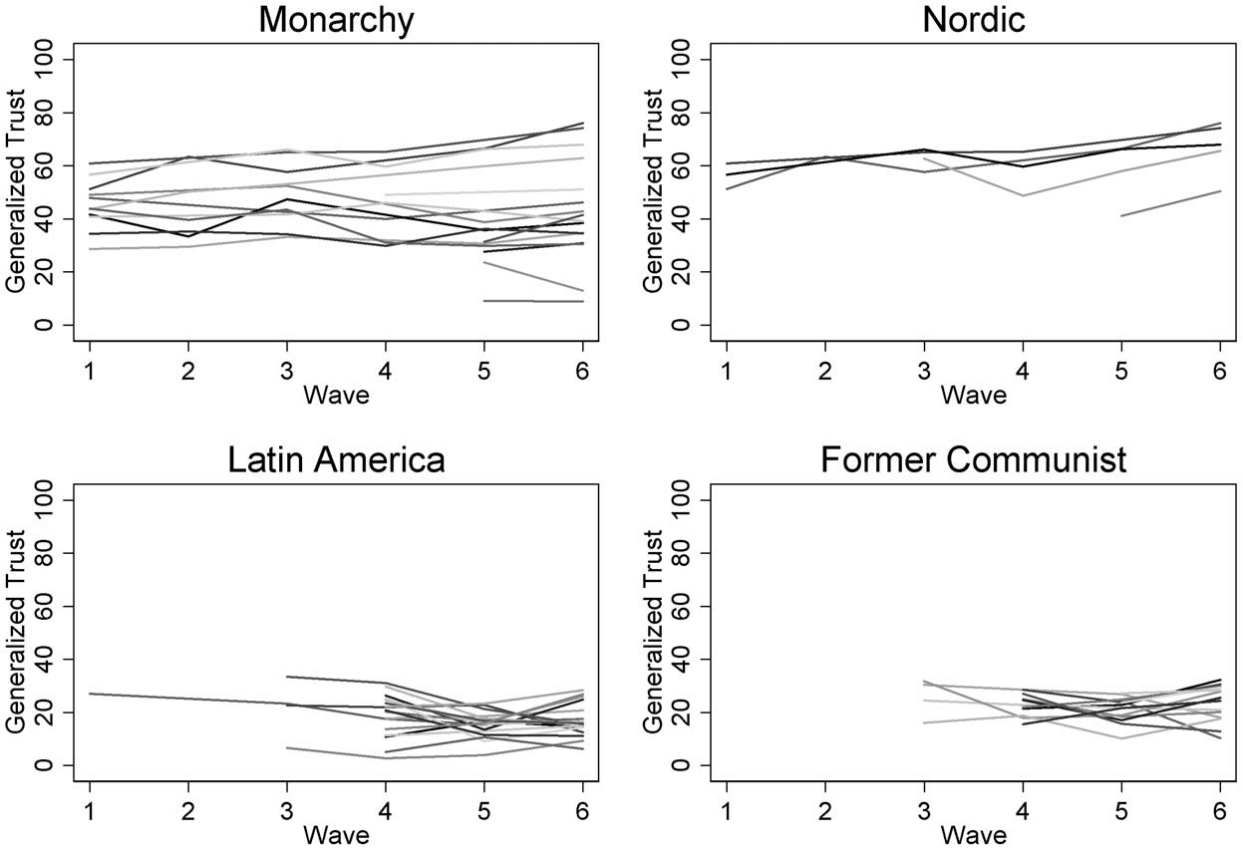
Generalized trust by monarchy, Nordic, Latin America, and former communist, 1980 to 2009.

Table S2 lists all generalized trust values by country and wave. The observations were abstracted from numerous cross-national public opinion data sets between 1980 and 2009. The unbalanced panel data are for 6 time periods with a total of 74 countries and 248 observations. See Table S2 for sources of generalized trust data. Replicating Roth [8], Table S2 highlights extensive variation within and between countries, with a strong decline in generalized trust for many countries across time. Figure 1 presents a plot of these temporal trends. The figure clearly shows that generalized trust has steadily increased for some countries (e.g., Denmark and Norway), while it has remained stable (e.g., Belgium, Brazil, and Ecuador) or steeply decreased for numerous others (e.g., Guatemala and Mexico). Figure 2 decomposes these temporal trends by country type. As expected, the figure shows that Nordic countries and monarchies exhibit much higher levels of generalized trust than either Latin American or former communist countries. Moreover, monarchies and Nordic countries generally reveal either growth or stability in generalized trust, while generalized trust in Latin American and former communist countries is either slowly growing or slowly decaying.

For all analyses, including the pooled-time series OLS estimates and the fixed- and random-effects estimates, we (a) exclude Greece, Iran, Nigeria, and Malawi as it is common in the literature to do so [8], [10] and since all four consistently exhibit extreme values across a number of outlier tests; (b) found that multicollinearity was only an issue for the property rights polynomial and that centering the two property rights terms did not substantively alter the results presented here–the variance inflation factor (i.e., VIF) for all other coefficients in the pooled-time series OLS models was well below (less than 4.0) the typical cut-off value of 10.0 [80]; (c) did not employ robust standard errors or robust-cluster standard errors by country since the Breusch-Pagan test for hetereoskedasticity revealed constant variance for all pooled time-series OLS models; and (d) provide one-tailed tests throughout.

### Pooled Time-series Analysis

**Figure 3:**
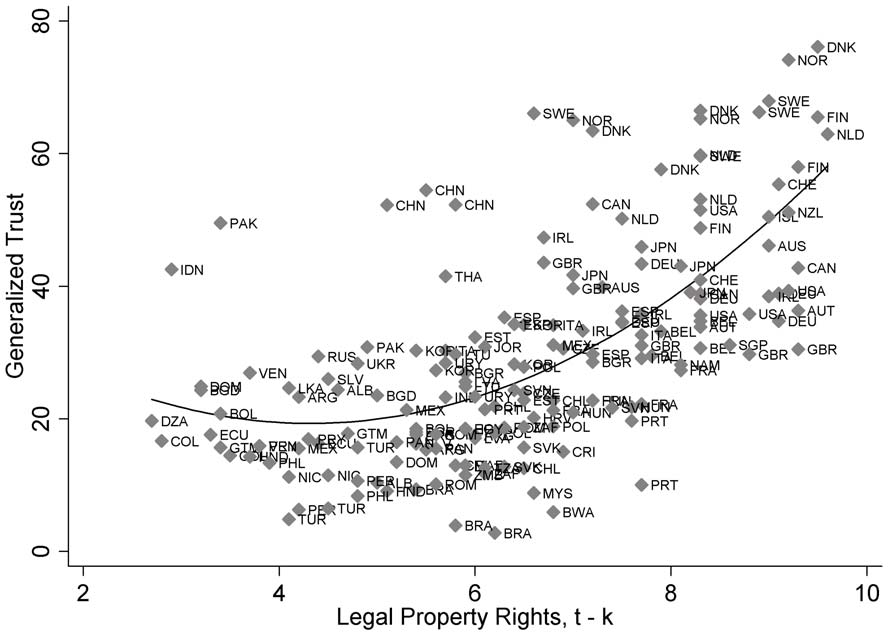
Legal property rights, _t–k_ and generalized trust, 1980 to 2009.

Table 2 presents a series of nested pooled time-series ordinary least squares regression models. Model 1 includes the key political institutional predictors and indicates that all variables, except for power-sharing capacity, have the expected signs and that only the legal property rights, state regulation, and size of government coefficients are statistically significant. As anticipated, increases in legal property rights undermine generalized trust at low levels of property rights protection. This negative effect, however, attenuates as the robustness of legal property rights increases. In other words, property rights institutions retard generalized trust in countries that have initial low levels of property rights protection but enhance generalized trust for countries that have fairly robust property rights institutions (see Figure 3). But note how the increasing effect of legal property rights on generalized trust is relatively much greater than the decreasing effect. This suggests that although legal property rights undermine generalized trust at low levels of property rights protection, the effect is relatively minor. Model 1 also shows that market deregulations and universal socioeconomic provisions promote generalized trust. Overall, the terms in model 1 do an excellent job of accounting for variance in generalized trust (R^2^ = .84) and tend to parallel prior results [19], [22], [42].

**Table 2:** Generalized trust and political institutions: A pooled panel analysis.

Parameters	Model 1	Model 2	Model 3	Model 4	Model 5
Generalized trust, _t–k_	.84*** (.05)	.81*** (.05)	.81*** (.05)	.81*** (.05)	.66*** (.06)
Legal property rights, _t–k_	−8.80*** (2.18)	−8.74*** (2.13)	−8.61*** (2.15)	−8.41*** (2.14)	−7.21*** (2.21)
Legal property rights^2^,_ t–k_	.71*** (.17)	.66*** (.17)	.66*** (.17)	.59*** (.17)	.53** (.18)
State regulation, _t–k_	1.51* (.73)	2.02** (.73)	1.98** (.74)	1.86** (.73)	1.37* (.74)
Worker’s rights, _t–k_	1.63 (1.00)	1.51 (.99)	1.55 (.99)	1.17 (1.00)	1.00 (.98)
Size of government, _t–k_	−1.11** (.42)	−.69 (.44)	−.71 (.44)	−.70 (.44)	−.24 (.46)
Power-sharing capacity, _t–k_	−1.38 (1.04)	−1.25 (1.02)	−1.22 (1.02)	−1.71 (1.04)	−1.57 (1.04)
Income inequality, _t–k_		−.19** (.07)	−.20** (.07)	−.21** (.07)	−.21** (.08)
Ethnolinguistic homogeneity, _t–k_			−.02 (.04)	−.04 (.04)	−.03 (.04)
ln(gross domestic product), _t–k_				1.52* (.78)	.82 (.84)
Monarchy					2.53* (1.52)
Nordic					7.36*** (2.29)
Temperature					−.11 (.09)
Pronoun-drop					.15 (1.67)
Former communist					−2.29 (2.02)
Constant	25.57** (8.61)	29.85** (8.58)	31.47*** (9.04)	23.72** (9.80)	28.74** (10.45)
R^2^	.84	.84	.84	.85	.86

Standard errors in parentheses.

* *p*<.05;

** *p*<.01;

*** *p*<.001 (one-tailed tests).

No. countries  = 74; No. observations  = 174.

Models 2 through 4 include nested controls for economic cleavages (i.e., income inequality), social cleavages (i.e., ethnolinguistic homogeneity), and modernization (i.e., gross domestic product), respectively. As expected, income inequality exhibits a statistically significant negative sign across all models and appears to mediate the relationship between size of government and generalized trust. Model 3 reveals the relationship between ethnolinguistic homogeneity and generalized trust to be statistically insignificant. Although this result appears to contradict prior research [14], [46], it parallels recent findings using larger samples [42], [72]. We also see that the relationship between GDP and generalized trust is statistically significant and positive, which supports Roth’s [8] pooled time-series OLS models exploring the relationship between economic growth and generalized trust. Besides size of government, all other political-institutional parameter estimates and standard errors are consistent from models 2 through 4.

Finally, model 5 includes the time-invariant controls–Monarchy, Nordic, Temperature, Pronoun-Drop, and Former Communist–and shows that the substantive story changes very little from model 4 to model 5 and that only the Monarchy and Nordic dummy variables are in the expected direction and statistically significant. All other time-invariant controls are statistically insignificant, which fails to support prior research [49], [72]. Interestingly, GDP becomes statistically insignificant when controlling for the time-invariant factors, some of which were absent from Roth’s [8] sensitivity analysis. Note that robust-cluster standard errors by country did not substantively alter the results presented here.

In short, the pooled time-series OLS analysis reveals the following: legal property rights and generalized trust are monotonically related–generalized trust increases at an increasing rate with legal property rights; government regulation of markets undermines generalized trust (higher values of state regulation indicate less market regulation); income inequality retards generalized trust; and Monarchies and Nordic countries are generally more trusting, on average, than other countries.

### Panel Analysis

In order to control for unobservable time-invariant factors (e.g., cultural history) and to explore how changes in predictors over time but not across countries affect generalized trust, we estimate a series of fixed- and random-effects panel models. As stated before, fixed-effects models estimate differences within countries while random-effects models estimate differences across countries as well as across time-periods. To test whether or not the variation across countries is correlated with the predictors in the models (i.e., independence assumption), we use the Hausman specification test [81]; the test indicates that fixed-effects estimation techniques should be used (the test statistic for models 7 and 8 in Table 3 is χ^2^ (10) = 91.46, which rejects the null hypothesis of independence). We nevertheless explore both fixed- and random-effects estimation for purposes of comparison. We also conducted joint tests on all fixed-effects models in Table 3 to see if dummy variables for time are equal to zero. The results suggest that time fixed effects are not needed. As before, we exclude Greece, Iran, Nigeria, and Malawi and use one-tailed tests throughout. We investigate alternative modeling specifications in our sensitivity analysis.

**Table 3:** Generalized trust and political institutions: fixed- and random-effects estimation.

Estimation method	FE	RE	FE	RE	FE	RE	FE	RE	RE
Parameters	Model 1	Model 2	Model 3	Model 4	Model 5	Model 6	Model 7	Model 8	Model 9
Generalized trust, _t–k_	.01 (.10)	.70*** (.06)	.01 (.10)	.67*** (.06)	.01 (.10)	.68*** (.06)	.01 (.10)	.67*** (.06)	.50*** (.07)
Legal property rights, _t–k_	−6.26* (2.79)	−8.49*** (2.32)	−6.26* (2.81)	−8.52*** (2.27)	−6.27* (2.83)	−8.53*** (2.28)	−7.21** (3.01)	−8.28*** (2.25)	−7.33*** (2.25)
Legal property rights^2^,_ t–k_	.44* (.20)	.68*** (.18)	.44* (.21)	.65*** (.18)	.44* (.21)	.65*** (.18)	.53* (.23)	.58*** (.18)	.52** (.18)
State regulation, _t–k_	1.33* (.71)	1.99** (.72)	1.32* (.74)	2.37*** (.73)	1.31* (.77)	2.37*** (.73)	1.46* (.79)	2.17** (.73)	1.63* (.72)
Worker’s rights, _t–k_	2.94** (1.17)	2.92** (1.06)	2.95** (1.18)	2.42** (1.04)	2.93** (1.19)	2.42** (1.05)	3.10** (1.21)	1.95* (1.05)	1.69* (1.02)
Size of government, _t–k_	−1.57* (.73)	−1.39*** (.49)	−1.57* (.74)	−.87* (.51)	−1.57* (.74)	−.87* (.51)	−1.40* (.77)	−.79 (.50)	−.28 (.52)
Power-sharing capacity, _t–k_	−6.48*** (1.57)	−2.18* (1.15)	−6.47*** (1.58)	−1.88* (1.12)	−6.46*** (1.59)	−1.88* (1.12)	−5.85*** (1.73)	−2.47* (1.15)	−2.32* (1.13)
Income inequality, _t–k_			.01 (.21)	−.23** (.08)	.01 (.21)	−.23** (.08)	.01 (.21)	−.23** (.08)	−.21* (.09)
Ethnolinguistic homogeneity, _t–k_					−.02 (.18)	.001 (.05)	−.05 (.18)	−.03 (.05)	−.02 (.05)
ln(gross domestic product), _t–k_							−3.55 (3.82)	2.02* (.95)	.83 (1.01)
Monarchy									4.33* (2.09)
Nordic									9.48** (3.26)
Temperature									−.17 (.11)
Pronoun-drop									−1.56 (2.33)
Former communist									−2.89 (2.67)
Constant	48.99***	26.16**	48.57***	32.17***	50.01*	32.07***	83.29*	20.87*	32.39**
R^2^ within	.23	.06	.23	.05	.23	.05	.24	.05	.06
R^2^ between	.01	.84	.01	.86	.01	.86	.11	.87	.86
σ_ν_	14.93	4.50	14.98	4.12	15.10	4.15	17.94	3.98	4.00
σ_e_	4.62	4.62	4.64	4.64	4.67	4.67	4.67	4.67	4.67
r	.91	.49	.91	.44	.91	.44	.94	.42	.42

Standard errors in parentheses.

* *p*<.05;

** *p*<.01;

*** *p*<.001 (one-tailed test).

No. countries  = 74; No. observations  = 174.

Table 3, like Table 2, presents a series of nested models but uses fixed- and random-effects estimation techniques instead. With the exception of Worker’s Rights and Power-Sharing Capacity, we see that the random-effects models (i.e., models 2, 4, 6, 8, and 9) parallel the pooled time-series ordinary least squares regression models found in Table 3. In other words, once we treat differences within- and between-countries across time as random variables and loosen the assumption of no unique attributes of countries and no universal effects across time, labor market regulations increase generalized trust and power-sharing capacities decrease generalized trust. The fixed-effects models, however, paint a different picture (see models 1, 3, 5, and 7): the results simultaneously support and contradict findings from prior empirical work and the pooled time-series OLS regression models in Table 3. Regardless of the fixed-effects model, all of the political-institutional variables are statistically significant. But unlike the OLS and random-effects models, income inequality is statistically unrelated to generalized trust once we time-demean the panel data (see models 3, 5, and 7). The fixed-effects estimates suggest that income inequality does not mediate the relationship between size of government and generalized trust. Gross domestic product is negatively related to generalized trust but statistically insignificant when using fixed-effects estimates (see model 7). Interestingly, when coupled with prior findings, this suggests that the causal relationship between economic growth and generalized trust runs not from growth to trust but from trust to growth. The interpretation for all statistically significant fixed-effects coefficients is as follows: for given country *i*, as *X* varies across time by one unit, generalized trust increases or decreases by *β* units. For instance, for given country *i*, as power-sharing capacity increases by one-unit across time, generalized trust decreases by 5.85 units (see model 7).

To explore alternative model specifications, we investigated whether or not the relationship between (a) gross domestic product and generalized trust and (b) power-sharing capacity and generalized trust were monotonic as argued by Roth [8] and as illustrated in a scatter plot, respectively. We did not find a statistically significant curvilinear relationship in our models for either specification (these findings held even when excluding legal property rights for both and gross domestic product for power-sharing capacity). Following Rothstein and Stolle [20] and Tsai et al. [42], we also explored a three-way interaction between legal property rights, power-sharing capacity, and worker’s rights. We introduced these terms to models 1 and 2 in Table 3. While the random-effects estimates replicated Tsai et al.’s [42] findings, the fixed-effects estimates did not. This suggests that their findings are likely an artifact of simultaneity or unobserved heterogeneity. We also explored interaction effects between ethnolinguistic homogeneity and migrant stock (as % of the population) in models 5 and 6, Table 3. Once again, the interaction effects were statistically insignificant. Results for all aforementioned alternative model specifications are available upon request.

### Sensitivity Analysis

The fixed-effects estimates appear to simultaneously support and challenge prior work using OLS, HLM, and 2SLS cross-sectional designs (e.g., [22], [49]) as well as cross-lagged structural equation panel models [18]. Since this is the case, we follow Wilson and Butler [82] and test the robustness and sensitivity of the results with respect to the key political-institutional variables. Table 4 reveals numerous specification tests that exclude possible influential cases, alter the countries included, investigate alternative control variables, restructure the panel data, and employ alternative model specifications. We use model 7 in Table 3 as the baseline model (see Row 1). Rows 2 through 4 alter the inclusion and exclusion of influential cases. As can be inferred, both the state regulations and size of government variables are sensitive to the inclusion of Greece, Iran, Nigeria, and Malawi (Row 2). This is expected as all four countries generate extreme values and, as a result, are considered influential cases that bias parameter estimates. While excluding China–a commonly excluded country in the generalized trust literature (e.g.,[10])–and other slightly influential cases such as Pakistan, Egypt, Thailand, Singapore, and Indonesia somewhat alter the size of the political-institutional coefficients, they all remain statistically significant (Rows 3 and 4, respectively). In short, the fixed-effects estimates for the political-institutional variables are fairly robust to the exclusion of influential cases.

**Table 4:** Sensitivity analysis: fixed-effects estimation.

Row		LPR	LPR^2^	SR	WR	SoG	PSC	N	Obser.
1	Baseline: Model 7, Table 3	−7.21***	.53**	1.46*	3.10***	−1.40*	−5.85***	74	174
Influential Cases									
2	With Greece, Iran, Nigeria, and Malawi	−4.94*	.38*	1.23	2.60**	−.76	−5.80***	78	181
3	No China	−7.23**	.53**	1.46*	3.11***	−1.44*	−5.86***	73	171
4	No China, Pakistan, Egypt, Thailand,Singapore, and Indonesia	−6.89**	.52**	1.40*	3.03***	−1.44*	−5.19***	68	165
Country Samples									
5	No Asia	−9.85***	.75***	1.51*	3.45***	−1.62*	−7.91***	60	147
6	No Eastern religions	−7.30**	.54**	1.46*	3.13***	−1.48*	−5.85***	69	164
7	No Africa	−7.29***	.53**	1.55*	3.12***	−1.42*	−5.85***	66	165
8	No Africa + Asia	−10.04***	.75***	1.59*	3.46***	−1.64*	−7.99***	52	138
9	No Latin America	−7.39	.51	1.74*	4.06***	−1.96**	−6.62***	56	133
10	No Nordic	−4.23	.26	1.07	2.73**	−1.08	−6.20***	69	159
11	No former communist	−8.05***	.56***	2.69**	2.19	−2.18**	−5.18***	60	144
12	No UN undeveloped	−11.29	.73	2.02	5.16***	−2.70***	−14.62***	35	101
13	No liberal	−8.98***	.77***	.99	2.59**	−.31	−5.08***	68	155
14	No conservative	−6.43**	.46*	1.36	3.03***	−1.05	−5.83***	68	152
15	No social democratic	−3.28	.17	.81	2.57**	−1.40*	−6.63***	68	153
16	No English legal origins	−8.99***	.77***	.93	2.38*	−.32	−4.53**	61	146
17	No French legal origins	−9.77	.66	1.62	4.40***	−1.87	−5.43*	39	92
18	No dictator since 1980	−10.09**	.69**	1.23	3.55***	−2.13***	−9.09***	56	136
19	No internal war last 10 years	−11.45***	.87***	1.08	2.98*	−1.43*	−3.28	59	138
20	No Protestant	−5.60*	.42*	1.05	2.65**	−.43	−5.55***	66	148
21	No Muslim	−7.69**	.55**	1.49*	2.77**	−1.16	−5.04***	64	159
Variable Specifications									
22	With migrant stock	−8.51**	.63**	1.92**	3.13**	−1.76**	−5.35***	73	169
23	With unemployment	−6.80**	.48**	1.61**	2.77**	−1.02	−5.85***	74	169
24	With religious homogeneity	−7.04**	.51**	1.54*	3.12**	−1.42*	−5.77***	74	174
25	With percent female	−7.26**	.54**	1.42*	3.06**	−1.42*	−5.85***	74	174
26	With tenure of political system	−9.66**	.75**	1.89**	2.92**	−1.46**	−5.36**	47	174
Restructuring of Data									
27	2 waves (bal.)	−6.39**	.50*	2.34***	1.82	.88	−5.08***	52	104
28†	3 waves (bal.)	3.03**	–	.73	1.26	−.25	−6.81*	17	51
29	Lagged variables, _t–1_	−7.37**	.61***	1.17	2.95***	−.84	−7.06***	72	153
Methods									
30	No generalized trust, _t–k_	−7.20***	.53**	1.45*	3.09***	−1.40*	−5.87***	74	174
31	Robust SE	−7.21**	.53**	1.46*	3.10***	−1.40	−5.85***	74	174
32	Bootstrap SE: 1,000 repetitions	−7.21**	.53**	1.46*	3.10***	−1.40	−5.85***	74	174
33	Jackknife	−7.21**	.53*	1.46	3.10***	−1.40	−5.85***	74	174
34	Wave dummies	−6.92*	.56**	1.34	2.69**	−.93	−5.16***	74	174
35	MVN multiple imp.: 20 Imput.	−6.37***	.49***	1.51**	2.94***	−.96	−5.05***	91	216
36	MVN multiple imp.: 100 Imput.	−6.47***	.50***	1.53**	3.00***	−.96	−4.98***	91	216
37‡	AR(1)	−9.30***	.67***	2.21***	1.45	−.72	−2.16*	74	174

† = Model 1, Table 3 FE estimation.

‡ = Model 8, Table 3 RE estimation.

* *p*<.05;

** *p*<.025;

*** *p*<.01 (one-tailed tests).

Rows 5 through 21 examine alternative country samples. Following Delhey et al. [66] and Torpe and Lolle [67], we explore the baseline model while excluding Asian and African countries (based on the United Nations categorization of Asian and African countries, see Row 5) and while excluding countries with majority populations practicing Eastern religions (see Row 6). This is done since individuals in Asian, African, and Confucian countries respond to the operationalization of generalized trust with a smaller radius in mind than individuals in other countries. Rows 5 through 8 shows that excluding these countries alone or in concert does not substantively alter the political-institutional fixed-effects estimates, while omitting Latin American and Nordic countries does (see Rows 9 and 10, respectively). Without Latin American countries, the legal property rights polynomial term becomes statistically insignificant, while the baseline model without Nordic countries yields insignificant effects for legal property rights, state regulations, and size of government. This suggests that the curvilinear effect for legal property rights is sensitive to Latin American and Nordic countries, which is intuitive since the upper-right quadrant of Figure 2 primarily consists of Nordic countries and the lower-left quadrant is primarily Latin American countries. In the absence of these countries, we would expect to see insignificant curvilinear coefficients for legal property rights. With respect to state regulations and size of government, both terms are nearly statistically insignificant, so it is intuitive that excluding countries with an emphasis on the universality of socioeconomic provisions *and* free-market competition would fuel the effects of both variables.

Next, we see that omitting former communist countries, liberal or conservative welfare states [we use Esping-Anderson’s [83] typology of welfare states–liberal (Australia, Canada, Ireland, New Zealand, United Kingdom, and United States), conservative (Finland, France, Germany, Italy, Japan, and Switzerland), and social-democratic (Austria, Belgium, Denmark, Netherlands, Norway, and Sweden)], countries with English legal origins, countries that have experienced any sort of dictatorship since 1980, countries that have experienced civil war within the last ten years of the survey year, and countries with Protestant or Muslim majority populations (i.e., 50% or more) did not substantively alter the statistical significance for the polynomial legal property rights term (see Rows 11 through 21). However, omitting undeveloped nations (as categorized by the United Nations Department of Economic and Social Affairs), social democratic welfare states, and countries with French legal origins did. Once again, this is the case as many of the social democracies are Nordic countries and many of the undeveloped and French legal origin countries are Latin American nations. The state regulations variable, on the other hand, is extremely sensitive to the exclusion of certain countries (except Latin American, Former Communist, and Muslim countries), while the worker’s rights and power-sharing capacity variables are very robust to the omission of cases (except Former Communist and Internal War, respectively). Size of government, like many of the other political institutional variables, appears to be sensitive to the omission of Nordic countries, welfare states, legal origins, and Protestant or Muslim majoritarian countries.

Rows 22 through 26 explore the robustness of the baseline model’s coefficients and standard errors to the inclusion of alternative control variables occasionally used in the literature [42], [48], [50]. We show that including World Bank data on either migrant stock (as % of the population), unemployment (as % of the labor force), or percent female (as % of total population) does not substantively alter the results for any of the political-institutional variables, except for size of government (see Row 23). Moreover, the findings are robust to religious homogeneity (as % largest religious group; from Britannica Book of the Year and CIA World Factbook) and tenure of political system (see Database of Political Institutions). Note: none of these control variables are statistically significant (not shown; results available upon request).

Rows 27 and 28 examine balanced panels models for the time periods 1999 to 2009 and 1994 to 2009, respectively. Despite balancing the data for 2 waves (see Row 27), many of the key political-institutional parameter estimates and standard errors parallel the baseline model: legal property rights increase generalized trust at an increasing rate, market deregulations increase generalized trust, and power-sharing capacity decreases generalized trust, while labor market regulations (i.e., worker’s rights) and the universality of socioeconomic provisions become statistically insignificant. Yet, when using a balanced panel with 17 countries and 51 observations over three waves, all key political-institutional variables lose statistical significance due to the small number of observations. But when we drop the polynomial term for legal property rights, the non-monotonic relationship between legal property rights and generalized trust becomes statistically significant as well as the coefficient for power-sharing capacity (see Row 28). We also see that using different time-series altered the statistical significance of some political-institutional predictors; Row 29 uses 1-period lagged variables and suggests that although the statistical significance for property rights, worker’s rights, and power sharing capacity remain unchanged, state regulations and size of government yield statistically insignificant coefficients.

Row 30 shows the baseline model without the *t–k* lagged generalized trust predictor. Doing so did not dramatically change the baseline model. Using robust standard errors (Row 31) and Bootstrap estimation with 1,000 repetitions (Row 32), however, rendered a statistically insignificant coefficient for size of government. Interestingly, both the state regulations and size of government variables became statistically insignificant with the use of Jackknife estimation (Row 33). And exploring time fixed-effects estimates with dummies for wave only altered the statistical significance of the state regulations and size of government parameters (Row 34).

Rows 35 and 36 examine the baseline model with imputation methods found in Stata12. The multiple imputation technique we use fills in missing values of the predictors and controls using multivariate normal regression with an iterative Markov Chain Monte Carlo (MCMC) method. We used a burn-in of 10,000 and calculated 1,000 MCMC iterations between imputations. The initial values of the MCMC chain were obtained from 1,000 expectation-maximum (EM) iterations. MCMC chain figures and plots revealed convergence. All missing values were imputed using predictors from model 5 in Table 2 plus dummies for wave, proportional representation, civil war after 1945, dictatorship since 1980, welfare-state typologies, regional location, and majoritarian religion. We also included continuous measures of the Herftot index (see Database of Political Institutions), tenure of political system (see Database of Political Institutions), and a country’s death rate (see World Bank). Finally, the relative variance increase (RVI) for the imputation models in Rows 35 and 36 were .22 and .23, respectively. Rows 35 and 36 specified 20 and 100 imputations to add, respectively. When comparing the baseline model to the imputation models, we see that although some of the coefficients changed in magnitude and that the number of countries increased from 74 to 91 and the number of observations increased from 174 to 216, respectively, the directions of effect and statistical significance of the coefficients remained unchanged except for the size of government coefficient.

Finally, Row 37 reveals the results of a first-order autoregressive random-effects model using the terms found in model 8, Table 3. We use random-effects estimation instead of fixed-effects estimation as the first-order autoregressive fixed-effects model yields inflated standard errors and directions of effect that deviate from the other fixed-effects models. This is the case as the number of countries and observations is reduced from 74 and 174 to 56 and 100, respectively. A small number of cases, observations, and panels can bias parameter estimates and standard errors when using first-order autoregressive fixed-effects modeling techniques [84]. Regardless, the standard errors are unbiased when comparing random-effects models with and without first-order autocorrelation (Model 8, Table 3 vs. Row 37, Table 4).

## Discussion

The political-institutional origins of generalized trust have long eluded social scientists. Although decades of research point to a possible association, no one study has provided convincing evidence for causal ordering–the ubiquity of cross-sectional designs and the use of theoretically invalid instrumental variables prohibit such conclusions. In an effort to advance the literature and address lingering issues of unobserved heterogeneity and simultaneity, the present study explores the relationship between political institutions and generalized trust using time-series panel analysis with a large cross-national sample of countries. In particular, we use pooled time-series OLS regression and fixed- and random-effects estimation techniques on a 74 country sample with a total of 248 observations spread over a 29 year time period (from 1980 to 2009) in order to test various competing theoretical models concerning the political-institutional foundations of generalized trust. Such an empirical investigation has yet to be done.

The paper, overall, reveals that political institutions simultaneously promote and undermine generalized trust. In particular, we find that property rights institutions are monotonically related to generalized trust: in countries with ineffective and inefficient property rights, an increase in their effectiveness leads to a decrease in generalized trust. This effect applies to undeveloped, developing, and Latin American countries. In countries with fairly effective and efficient property rights, an increase in their effectiveness leads to an increase in generalized trust (especially in social democratic and Nordic countries). Interestingly, this effect is one of the least sensitive of the political-institutional factors to the restructuring of data, statistical modifications, countries included (or excluded), influential cases, and confounding control variables.

Our analysis provides support for the argument that political-institutional incentives foster expectations and beliefs about the reliability of anonymous others, and that effective legal structures and property rights institutions create an environment where common knowledge about the trustworthiness of strangers can grow. Although the results suggest that property rights undermine generalized trust at low levels of protection, the increasing effect of legal property rights on generalized trust is relatively much greater than its undermining effect at low levels of contract enforcement. All of which buttress Bohnet, Frey, and Huck’s experimental findings: “The worst legal regime is not one in which contracts cannot be enforced but one with an intermediate level of enforceability” [69]. Strictly speaking, regimes with intermediate levels of property rights protection neither adequately prosecute malfeasance nor leave the guarantee of exchanges solely to decentralized systems of informal regulation: both of which foster generalized trust. The former is typical of regimes with high levels of contract enforcement, while the latter is characteristic of regimes with low levels contract enforcement, neither of which is robust with intermediate levels of property rights protection.

Second, we show that market deregulations are positively related to generalized trust. The finding supports the argument that economic, business, and credit markets regulated by government appointed bodies weakens community building, civil society, and generalized trust [22]. As Taylor [31] suggests, the likely mechanism accounting for this effect is dependence: individuals come to depend on government bodies instead of each other in order to promote social and economic exchange in the presence of centralized market regulations, thereby diminishing generalized trust. This effect, however, appears to be fueled by Scandinavian cultural legacies, welfare state typologies, legal origins, and internal strife, and is also sensitive to the restructuring of data and resampling techniques. In other words, the relationship between market deregulations and generalized trust found in the present study and prior research [22] is likely a statistical artifact.

Third, unlike economic market regulations, increases in labor market regulations increase generalized trust. This supports Bendix’s [33] and Marshall’s [34] classic proposition: government bodies that regulate labor markets, such as minimum wage laws for instance, expand the rights and privileges of citizens, which promotes nationalistic social bonds and social unification and integration that, in turn, increases generalized trust. Yet we find that former communist countries, restructured panel data, and autocorrelation seem to fuel this effect. In spite of this, the labor market regulations variable is one of the political-institutional factors least sensitive to sample adjustments, statistical modifications, resampling techniques, confounding control variables, and influential cases.

Fourth, in contrast to prior research [18], power-sharing capacity of the state is negatively related to generalized trust: as states increasingly share and divide their power–as states democratize–generalized trust decreases. Embedded in this finding is an implicit refutation of the merits of democracy and an explicit confirmation of the idea that democratic governments represent the special interests of parties [20]. Such regimes create governance structures biased towards certain interests and unresponsive to the needs of all citizens, resulting in weakened generalized trust especially among disadvantaged and excluded groups. More broadly interpreted, the deleterious impact of partisan politics on generalized trust is, contrary to Lipset [85], unavoidable in a stable democracy. Yet an alternative interpretation of the results concerns not the special interests of political parties *per se*, but the process of democratization: transitions to democracy might damage community structures that help foster social capital and generalized trust under institutional conditions incompatible with democracy [79], [86]. In this approach, the adaptation to new political-institutional environments and surroundings that result from democratization is what weakens generalized trust, not the catering to special interests of political parties. What these two competing arguments suggest is the need for future research to explicitly disentangle the exact mechanisms connecting power-sharing capacity to generalized trust, which we find to be the least sensitive of the political-institutional factors to any sort of model re-specifications. Only by omitting countries that have experienced civil war and internal strife within the last 10 years of the survey year (e.g., Algeria and Indonesia) does the coefficient for power-sharing capacity become statistically insignificant.

Fifth, our models support prior research [19], [35], [36] and show that the universality of socioeconomic provisions is related to generalized trust. To illustrate, universal political institutions, such as welfare states, foster economic equality, equality of opportunity, and evenly distributed resources. These types of regimes create the perception that each citizen has an equal opportunity of success and failure, regardless of their class, gender, or ethnicity, and that no one economic actor is more privileged than another; as a consequence, social cleavages and social barriers break down, while community building, social cohesion, and social solidarity grow. All of which should increase generalized trust. Yet we find the standard errors for size of government–our measure of universality–to be extremely sensitive to statistical modifications, countries included or excluded, and confounding control variables. Moreover, when using either a pooled time-series OLS or a random-effects panel design, we replicate the negative relationship between income inequality and generalized trust [10]. But when we examine the same sample with fixed-effects estimates, we fail to detect a statistically significant relationship. What all of this suggests is that theorists and policy analysts should seriously reconsider the effects that welfare states and income inequality have on generalized trust in particular and social capital in general.

To summarize, what appears to matter for generalized trust is the safety and security granted by property rights institutions, the centralized regulation of labor markets, and stable regimes that avoid sudden shifts toward democratization. In contrast, sources of generalized trust are not found with the deregulation of credit and business markets or the universality of socioeconomic provisions as these political-institutional factors are extremely sensitive to sample adjustments, statistical modifications, resampling techniques, and influential cases. Overall, the findings generate strong support for various competing theoretical models within the social sciences: some models suggest that political institutions undermine generalized trust, while others argue that political institutions create an environment where generalized trust can grow. We provide evidence for both–political institutions crowd-in and crowd-out generalized trust. In spite of the results, future research should explore the specific elements of legal property rights institutions and labor market regulations that promote or possibly undermine generalized trust–such as judicial independence, impartial courts, private ownership of banks, private sector credit, and conscription–and investigate panel-data with two-stage least squares when theoretically and empirically valid instrumental variables for political institutions are available.

## Supporting Information

Table S1 **Sources of variables.**
(DOC)

Table S2 **List of cross-sectional time-series values for generalized trust.**
(DOC)
