# Supplementary material for: A Blessing and a Curse? Political Institutions in the Growth and Decay of Generalized Trust: A Cross-National Panel Analysis, 1980–2009
Source: PLoS One. 2012 Apr 25;7(4):e35120. doi: 10.1371/journal.pone.0035120 (PMC3338835; doi:10.1371/journal.pone.0035120)
Supplement: Table S1 — Sources of variables. (DOC) [file pone.0035120.s001.doc]

| **Table S1.** Sources of variables. | | | | | | | |
| --- | --- | --- | --- | --- | --- | --- | --- |
| Variables | Source |  |  |  |  |  |  |
| Generalized trust | WVS, EVS, and various barometers from 1980 to 2009. | | | | | | |
| Legal property rights | Economic Freedom of the World Project, Legal Structure and Security of Property rights. | | | | | | |
| State regulations | Economic Freedom of the World Project, Regulation of Credit, Labor, and Business. | | | | | | |
| Worker's rights | Cingranelli and Richards (2008). | | | | | | |
| Size of government | Economic Freedom of the World Project, Size of Government: Expenditures, Taxes, Enterprises. | | | | | | |
| Power-sharing capacity | See below (political rights, power-sharing regime, democracy, and executive authority). | | | | | | |
| Political rights | United Nations, Freedom House web resources. | | | | | | |
| Power-sharing regime | Norris (2008). | | | | | | |
| Democracy | Polity IV Project, Political Regimes 1800-2009. | | | | | | |
| Executive authority | Polity IV Project, Political Regimes 1800-2009. | | | | | | |
| Income inequality | World Bank, OECD, CIA World Factbook, and Earth Trends. | | | | | | |
| Ethnolinguistic homogeneity | Britannica Book of the Year and CIA World Factbook. | | | | | | |
| Gross domestic product | World Bank. | | | | | | |
| Monarchy | Absolute or Constitutional Monarchies. | | | | | | |
| Nordic | Denmark, Finland, Iceland, Norway, and Sweden. | | | | | | |
| Temperature | Average Coldest Month of the Year (see World Meteorological Organization). | | | | | | |
| Pronoun-drop | License to Pronoun-Drop in a Country's Official Language (see Kashima and Kashima 1998). | | | | | | |
| Former communist | Former Marxist-Leninist Communist States. | | | | | | |
