# Supplementary material for: A Blessing and a Curse? Political Institutions in the Growth and Decay of Generalized Trust: A Cross-National Panel Analysis, 1980–2009
Source: PLoS One. 2012 Apr 25;7(4):e35120. doi: 10.1371/journal.pone.0035120 (PMC3338835; doi:10.1371/journal.pone.0035120)
Supplement: Table S2 — List of cross-sectional time-series values for generalized trust. (DOC) [file pone.0035120.s002.doc]

| **Table S2**. List of cross-sectional time-series values for generalized trust. | | | | | | |
| --- | --- | --- | --- | --- | --- | --- |
|  | Wave | | | | | |
|  | I | II | III | IV | V | VI |
| Country | 1981-1984 | 1986† | 1989-1993 | 1994-1998 | 1999-2004 | 2005-2009 |
| Albania |  |  |  | 27 | 24 | 10‡ |
| Algeria |  |  |  |  | 11 | 20ǂ |
| Argentina | 27 |  | 23 | 18 | 15 | 18 |
| Australia | 48 |  | 40 |  |  | 46 |
| Austria |  |  | 32 |  | 34 | 36‡ |
| Bangladesh |  |  |  | 21 | 24 | 24ǂ |
| Belgium | 29 | 30 | 33 |  | 31 | 35‡ |
| Bolivia |  |  |  | 18ǂ | 19ǂ | 21ǂ |
| Botswana |  |  |  |  | 15ǂ | 6ǂ |
| Brazil |  |  | 7 | 3 | 4ǂ | 9 |
| Bulgaria |  |  | 30 | 29 | 27 | 18‡ |
| Canada | 49 |  | 52 |  | 39 | 43 |
| Chile |  |  | 23 | 22 | 23 | 13 |
| China |  |  | 60 | 52 | 55 | 52 |
| Colombia |  |  |  | 11 | 17ǂ | 14 |
| Costa Rica |  |  |  | 11ǂ | 13ǂ | 15ǂ |
| Croatia |  |  |  | 25 | 18 | 20‡ |
| Czech Republic |  |  |  | 29 | 24 | 31‡ |
| Denmark | 51 | 64 | 58 |  | 67 | 76‡ |
| Dominican Republic |  |  |  | 26 | 13ǂ | 25ǂ |
| Ecuador |  |  |  | 20ǂ | 16ǂ | 18ǂ |
| Egypt |  |  |  |  | 38 | 19 |
| El Salvador |  |  |  | 25ǂ | 16ǂ | 26ǂ |
| Estonia |  |  |  | 22 | 23 | 32‡ |
| Finland |  |  | 63 | 49 | 58 | 66‡ |
| France | 24 | 21 | 23 |  | 22 | 27‡ |
| Germany | 31 | 43 | 35 | 38 | 35 | 39‡ |
| Guatemala |  |  |  | 30ǂ | 18ǂ | 16 |
| Honduras |  |  |  | 25ǂ | 9ǂ | 14ǂ |
| Hungary |  |  | 25 | 23 | 22 | 21‡ |
| Iceland |  |  |  |  | 41 | 50‡ |
| India |  |  |  |  | 41 | 23 |
| Indonesia |  |  |  |  | 52 | 43 |
| Ireland | 42 | 33 | 47 |  | 36 | 38‡ |
| Italy | 25 | 30 | 34 |  | 33 | 29 |
| Japan | 41 |  | 42 | 46 | 43 | 39 |
| Jordan |  |  |  |  | 28 | 31 |
| Latvia |  |  |  | 25 | 17 | 26‡ |
| Lithuania |  |  |  | 22 | 25 | 30‡ |
| Malaysia |  |  |  |  | 9 | 9 |
| Mexico |  |  | 33 | 31 | 21 | 16 |
| Morocco |  |  |  |  | 24 | 13 |
| Namibia |  |  |  |  | 35ǂ | 28ǂ |
| Netherlands | 44 | 50 | 53 |  | 60 | 63‡ |
| New Zealand |  |  |  | 49 |  | 51 |
| Nicaragua |  |  |  | 21ǂ | 11ǂ | 11ǂ |
| Norway | 61 |  | 65 | 65 |  | 74 |
| Pakistan |  |  |  | 21 | 31 | 50ǂ |
| Panama |  |  |  | 25ǂ | 16ǂ | 16ǂ |
| Paraguay |  |  |  | 24ǂ | 17ǂ | 16ǂ |
| Peru |  |  |  | 5 | 11 | 6 |
| Philippines |  |  |  | 6 | 8 | 13ǂ |
| Poland |  |  | 32 | 18 | 19 | 28‡ |
| Portugal |  | 28 | 21 |  | 10 | 20‡ |
| Romania |  |  | 16 | 24 | 10 | 18‡ |
| Russian Federation |  |  |  |  | 24 | 29‡ |
| Singapore |  |  |  |  | 17 | 31ǂ |
| Slovakia |  |  |  | 27 | 16 | 13‡ |
| Slovenia |  |  |  | 16 | 22 | 24‡ |
| South Africa |  |  |  | 18 | 12 | 19 |
| South Korea | 38 |  | 34 | 30 | 27 | 28 |
| Spain | 34 | 35 | 34 | 30 | 36 | 35‡ |
| Sri Lanka |  |  |  |  | 11ǂ | 25ǂ |
| Sweden | 57 |  | 66 | 60 | 66 | 68 |
| Switzerland |  |  | 43 | 41 |  | 55‡ |
| Tanzania |  |  |  |  | 8 | 13ǂ |
| Thailand |  |  |  |  | 31 | 42 |
| Turkey |  |  | 10 | 7 | 16 | 5 |
| Ukraine |  |  |  |  | 27 | 28‡ |
| United Kingdom | 44 | 40 | 44 | 31 | 30 | 31 |
| United States | 40 |  | 52 | 36 | 36 | 39 |
| Uruguay |  |  |  | 22 | 23ǂ | 28 |
| Venezuela |  |  |  | 14 | 16 | 27ǂ |
| Zambia |  |  |  |  | 20ǂ | 12 |
| WVS/EVS integrated data unless specified. | | | | | | |
| † = 1986 Eurobarometer 25 data. | | | | | | |
| ǂ = Asia, Latino, or Afro barometer data. | | | | | | |
| ‡ = 2008 EVS Wave IV data. | | | | | | |
| No. countries = 74; No. observations = 248. | | | | | | |
